# Intra-ocular melanoma metastatic to an axillary lymph node: A case report

**DOI:** 10.1186/1477-7819-9-61

**Published:** 2011-05-27

**Authors:** Nirupama Anne, Ratnakishore Pallapothu

**Affiliations:** 1 Department of Surgery, Our Lady of Lourdes Memorial Hospital, Binghamton, NY, USA

## Abstract

**Background:**

Unusual metastatic presentation of intra-ocular melanoma.

**Study Design:**

Case report.

**Discussion:**

Extra-regional lymphatic spread of intra-ocular melanoma has not been reported previously in the literature. The usual pattern of metastasis for intra-ocular melanoma is hematogenous. There are few reports of regional spread to the maxillofacial bones. We report an interesting case of a 51 year old female with prior history of right eye melanoma, now presenting with metastasis to the left axilla, which is an extra-regional nodal basin.

**Conclusion:**

In female patients presenting with an isolated axillary mass, with a negative breast work up and known prior history of melanoma, the differential diagnosis should include possible metastatic melanoma. Core biopsy will confirm the diagnosis and tailor subsequent management.

## Introduction

Ocular melanoma is the most common type of eye cancer among adults followed by intra-ocular lymphoma. Melanoma develops from pigment producing cells called melanocytes. 90% of the intra-ocular melanomas develop in the choroid (which is part of the uvea). The etiology is unknown. There are studies to indicate the role of sunlight or artificial exposure to ultra-violet radiation (UVR), but the evidence is mixed [1,2]. Regional lymph node metastasis from choroidal melanoma is extremely rare. Here we report an unusual case of a lady diagnosed with choroidal melanoma metastatic to an axillary lymph node. Reports of metastasis to extra-regional lymph node basins such as the axilla have not been reported thus far based upon our review of the literature which makes this case unique.

## Case Report

A 51 year old Caucasian lady presented to the breast care center with two week duration of left axillary mass. No other breast symptoms. Past medical history is significant for right eye choroidal melanoma diagnosed 1.5 years ago treated with brachytherapy and followed at an eye institute.

At the time of her diagnosis, the patient was having right eye visual field defect which prompted the evaluation, and the melanoma was noted to be 16 mm in diameter with 9.3 mm thickness, choroidal location, with inferior hemi-retinal detachment. She is still under follow-up care from the eye institute with clinical response to the brachytherapy treatment. She had a dermatologic examination of the whole body to document no cutaneous sites of concern. Family history is significant for her father, paternal aunt, and paternal first cousin who were diagnosed with cutaneous melanoma and underwent treatment.

**Figure 1:**
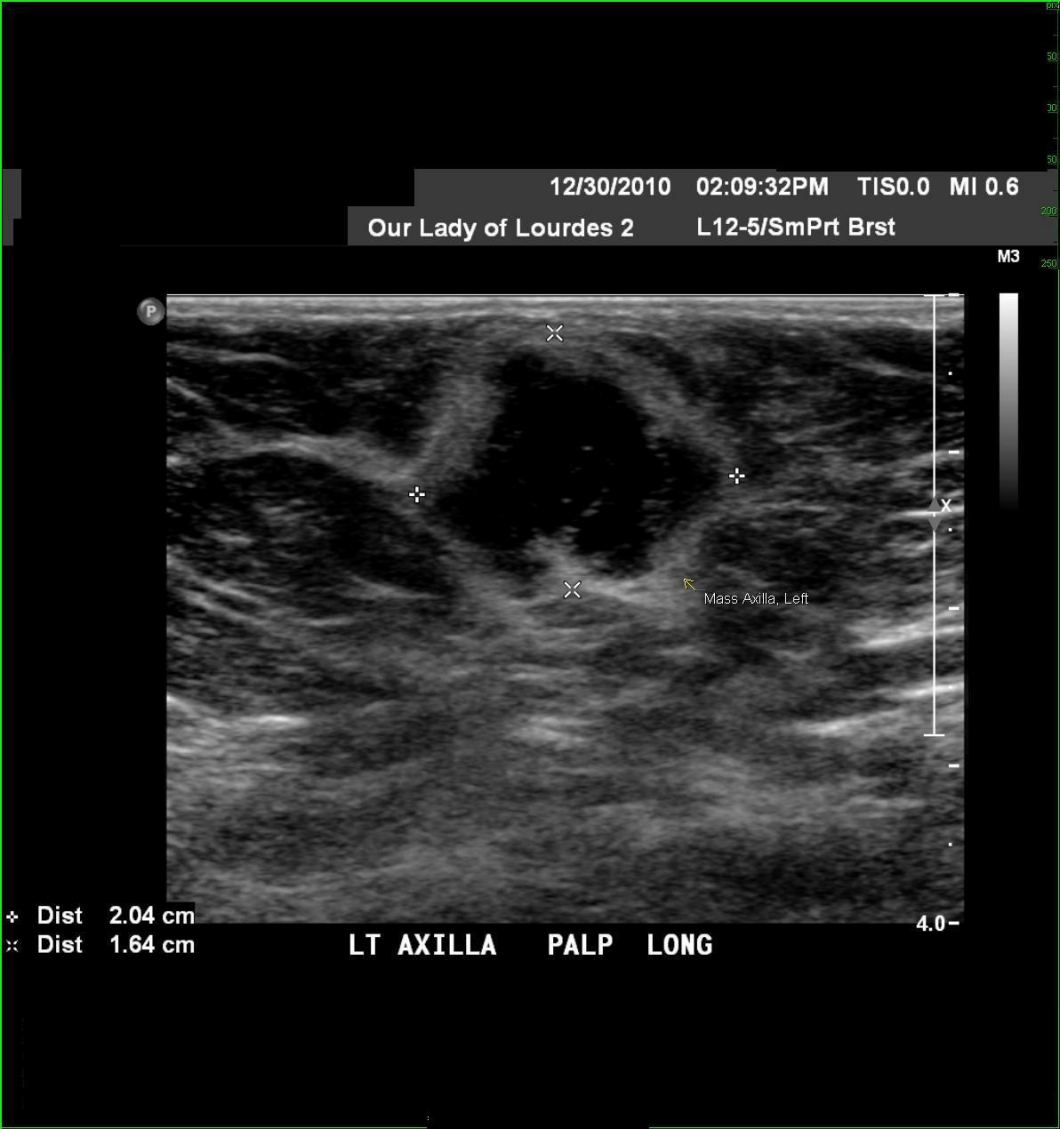
**Ultrasound image of the irregular mass in the left axilla**.

Physical examination was within normal limits with the exception of the left axilla where there is a 2 cm × 2 cm, freely mobile, non-tender, lymph node. Mammograms from three weeks prior were within normal limits. Ultrasound of the left axilla done a week prior to the evaluation (Figure 1) showed an irregular mass, 2.0 × 1.6 × 2.0 cm in size, hypo-echoic, heterogeneous, with some peripheral blood flow. No edge artifact, no posterior acoustic enhancement or shadowing consistent with BIRADS 4 imaging.

**Figure 2:**
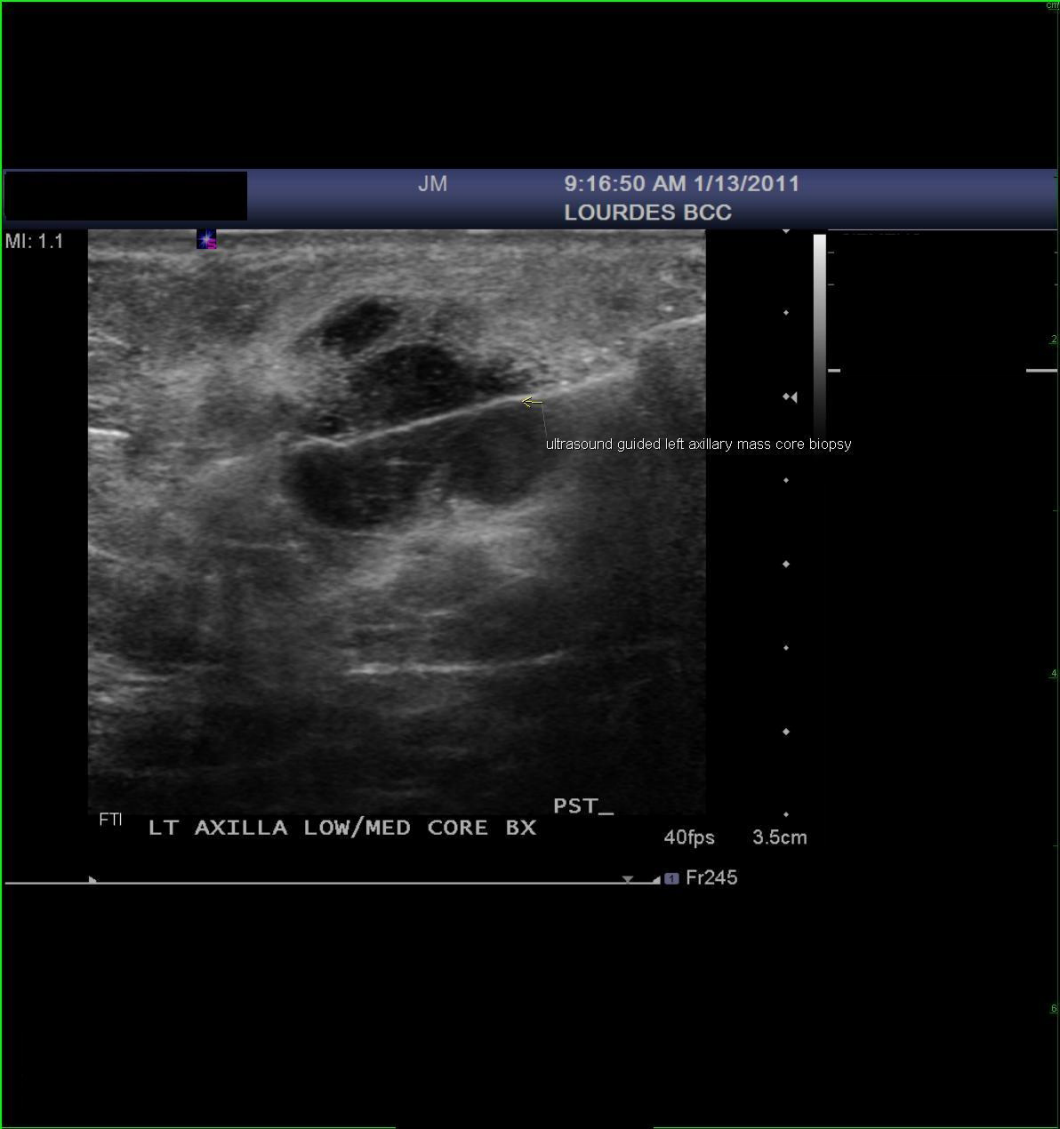
**The image shows the ultrasound guided core biopsy of the left axillary mass**.

**Figure 3:**
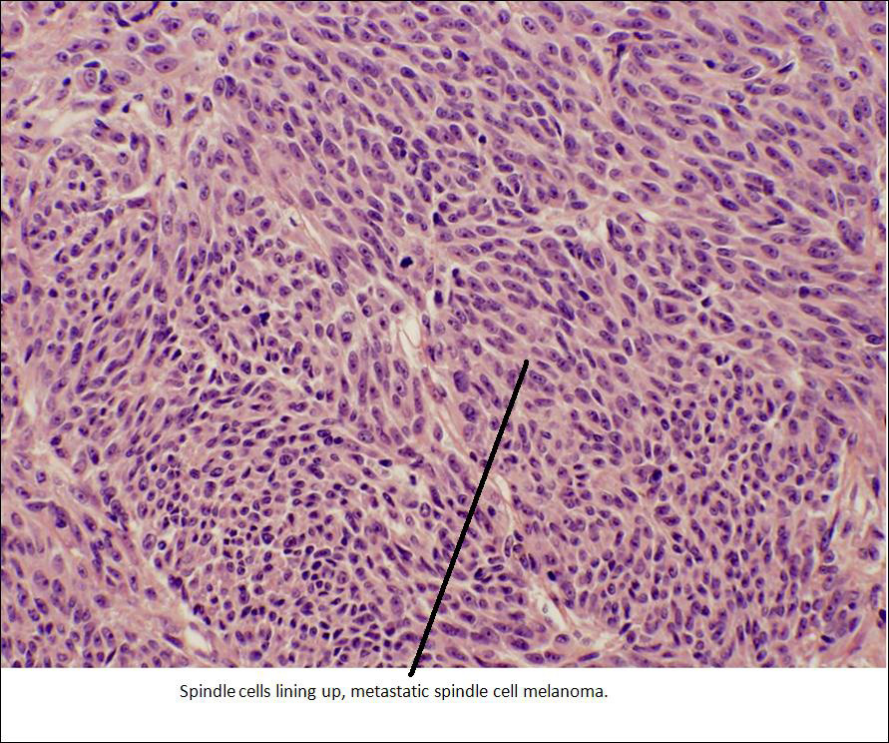
**Histopathology image of the core biopsy showing metastatic spindle cell melanoma**.

Subsequently, the patient underwent an ultrasound guided left axillary mass core biopsy (Figure 2). Pathology on the core biopsy demonstrated metastatic spindle cell melanoma with necrosis (Figure 3). The patient underwent extensive staging workup including a PET/CT scan which showed a single site of hypermetabolic activity along the left mid-axillary line in the axilla. There was resolution of anatomic findings related to the right orbit (initial site of melanoma) and no adenopathy elsewhere. The solid organs were within normal limits. She was referred to an NCI designated tertiary Institute for a consultation regarding clinical trials for systemic therapy involving interferon based versus surgery and observation.

## Discussion

The incidence of intra-ocular melanomas has been stable over the last 25 years, at 6 cases per1 million population [1-8]. Risk factors for intra-ocular melanoma include Caucasian race, light skin and or eye color, dysplastic nevus syndrome, oculo-dermal melanocytosis (nevus of Ota), sun exposure, occupation exposure (welders, chemical workers). The etiology for the most part is multi-factorial or unknown [2].

Most patients with melanoma of the eye do not have symptoms. Symptoms however can include blurry vision, loss of vision, floaters, visual field loss (as in our patient), growing dark spot on the iris, alteration in the size or shape of the pupil, change in the position of the eyeball, bulging of the eye, change in eye movements, and light sensitivity. Pain is a very rare symptom [2,3].

Most of the time a comprehensive eye exam alone by an Ophthalmologist can make the diagnosis [4]. Rarely an ultrasound or a biopsy is needed. Intra-ocular melanomas are generally made up of two different kinds of cells namely, spindle (long, thin cells) and epitheloid (round, straight) cells. Most tumors are composed of both kinds of these cells. Epitheloid tumors are more likely to metastasize to distant sites than spindle cell variant (which is the histology in this case). The mode of metastasis is hematogenous for both histological subtypes, with the first site being the liver [3,4]. Tumor size is a significant prognostic factor for the development of metastatic disease [3-6]. Extra-ocular spread to other organs such as lung, gastrointestinal tract, skin, bones, central nervous system, has been seen in association with liver metastases [5,6].

There are very few case reports of regional lymph node metastasis from an intra-ocular melanoma. These studies reported spread of choroidal melanoma into the conjunctiva via regional lymphatics [5] and or spread to the maxillofacial bones [6]. Extra-ocular distant lymphatic spread (outside the regional lymph node basin) has not been demonstrated in intra-ocular choroidal melanomas due to the absence of lymphatics in the choroid. There is some research and speculation on intraocular lymphangiogenesis in melanomas of the ciliary body and if that could explain extra-ocular lymph node spread or extension [7]. The case we present is unusual as it demonstrates lymphatic spread of choroidal melanoma outside the eye to an extra-regional lymph node basin which has not been reported previously in the literature.

Prognosis of intra-ocular melanoma depends upon the stage of the disease. Staging for melanoma of the eye differs from cutaneous melanoma. Furthermore melanoma involving the iris has a separate T staging than the melanoma involving the ciliary body/choroidal plexus. Cancer spread involving different parts of the body, like the scenario in this case, is Stage IV. Survival rate for patients with Stage IV melanoma at 5 years is approximately 15% [8,9].

Surgical therapy of choroidal melanoma traditionally involves enucleation. Brachytherapy, also known as episcleral plaque therapy, can be used as a primary treatment modality. Some studies have shown that in many cases it is as effective as enucleation [8,9].

## Conclusion

Most melanomas of the eye involve the choroid. The diagnosis is often clinically made by an Ophthalmologist. The pattern of metastatic spread has been traditionally thought to be hematogenous, liver being the first site. This case illustrates that intra-ocular melanoma has the potential to metastasize to extra-ocular distant lymphatic basin. Unusual metastasis poses a diagnostic and therapeutic challenge.

## Competing interests

Nirupama Anne, MD: Myriad Genetics Laboratory, Local Speaker.

Ratnakishore Pallapothu, MD: None.

## Authors' contributions

NA contributed to the collection of the clinical data and writing of the manuscript. RP contributed to the writing and editing of the manuscript. Both authors read and approved the final manuscript.
